# Establishing a deeper understanding of the osteogenic differentiation of monolayer cultured human pluripotent stem cells using novel and detailed analyses

**DOI:** 10.1186/s13287-020-02085-9

**Published:** 2021-01-07

**Authors:** Ping Zhou, Jia-Min Shi, Jing-E Song, Yu Han, Hong-Jiao Li, Ya-Meng Song, Fang Feng, Jian-Lin Wang, Rui Zhang, Feng Lan

**Affiliations:** 1 School and Hospital of Stomatology, Lanzhou University, No.222 Tianshui South Road, Chengguan District, Lanzhou, 730000 Gansu Province People’s Republic of China; 2 College of Life Sciences, Lanzhou University, No.222 Tianshui South Road, Chengguan District, Lanzhou, 730000 Gansu Province People’s Republic of China; 3 National Center for Cardiovascular Diseases, Fuwai Hospital, Chinese Academy of Medical Sciences and Peking Union Medical College, Beijing, 100037 People’s Republic of China

**Keywords:** Osteogenic differentiation, Human embryonic stem cells, Human-induced pluripotent stem cells, Marker expression

## Abstract

**Background:**

Derivation of osteoblast-like cells from human pluripotent stem cells (hPSCs) is a popular topic in bone tissue engineering. Although many improvements have been achieved, the low induction efficiency because of spontaneous differentiation hampers their applications. To solve this problem, a detailed understanding of the osteogenic differentiation process of hPSCs is urgently needed.

**Methods:**

Monolayer cultured human embryonic stem cells and human-induced pluripotent stem cells were differentiated in commonly applied serum-containing osteogenic medium for 35 days. In addition to traditional assays such as cell viability detection, reverse transcription-polymerase chain reaction, immunofluorescence, and alizarin red staining, we also applied studies of cell counting, cell telomerase activity, and flow cytometry as essential indicators to analyse the cell type changes in each week.

**Results:**

The population of differentiated cells was quite heterogeneous throughout the 35 days of induction. Then, cell telomerase activity and cell cycle analyses have value in evaluating the cell type and tumourigenicity of the obtained cells. Finally, a dynamic map was made to integrate the analysis of these results during osteogenic differentiation of hPSCs, and the cell types at defined stages were concluded.

**Conclusions:**

Our results lay the foundation to improve the in vitro osteogenic differentiation efficiency of hPSCs by supplementing with functional compounds at the desired stage, and then establishing a stepwise induction system in the future.

## Background

Large-area bone defects are hard to treat in the clinic owing to the limited regenerative capability of bone tissues. Moreover, existing therapeutic methods exhibit problems such as immunity risk, surgical trauma and ethical confusion. Recently, researchers have considered tissue engineering technology to solve this problem, benefiting from significant progress having been made in growth factors and scaffolds [1]. However, how to obtain a large number of functional osteoblast cells from stem cells is a huge barrier to achieving good results of large bone regeneration in animal models [2]. Traditionally, mesenchymal stem cells (MSCs) have been popularly used to derive osteoblast-like cells. However, the number of obtained cells cannot meet the demand because of challenges that include limited source, bad batch stability and aging of the cells [3]. Fortunately, human embryonic stem cells (hESCs) and human-induced pluripotent stem cells (hiPSCs) harbour unique long-term self-renewal and multi-directional differentiation potential, and they are undoubtedly the preferred seed cell origin for bone tissue engineering [4].

In recent decades, many researchers have derived osteoblast-like cells from human pluripotent stem cells (hPSCs). However, the step by step differentiation involving mesoderm or ectoderm cells, mesenchymal-like cells and finally osteoblast-like cells is far from established. The results in the efficiency of osteogenic differentiation of hPSCs using current methods are much lower than that of cell induction into cardiomyocyte-like cells, neuron-like cells and hepatocytes [5]. Up to now, a medium consisting of foetal bovine serum (FBS), dexamethasone, β-glycerophosphate and vitamin C (ascorbic acid) is still commonly used for the osteogenic differentiation of hPSCs. To establish an in vitro directed induction system, many functional chemical compounds should be supplied at specific differentiation stages for the serial induction of the abovementioned cells. Therefore, it is necessary to understand the dynamic changes of markers and cell types during the osteogenic differentiation of hPSCs, which helps to judge the optimal supplementary period for osteogenic induction factors and enhance the efficiency of osteogenesis.

To date, many analyses including staining and expression detection of marker genes or proteins have been explored to identify and monitor the osteogenic differentiation process. Specifically, alizarin red and von Kossa staining are used to detect the deposition of calcium nodules in cells. Then, alkaline phosphatase (ALP) staining and BCIP/NBT colorimetry are applied to evaluate the ALP activity. More critical, pluripotency-related markers of OCT-4 and NANOG, as well as many osteogenesis-related makers such as ALP, runt-related transcription factor 2 (RUNX2), osterix (OSX), type I collagen (COL1A1), osteocalcin (OCN), bone sialoprotein (BSP) and osteopontin (OPN), were detected using molecular biology techniques. Obviously, the analyses that are applied to clarify the osteogenic differentiation process of hPSCs are almost the same as those for MSCs. It has been reported that hPSCs induced into osteoblasts undergo proliferation, differentiation, deposition of extracellular matrix and mineralization [6]. However, few systematic studies have been performed to analyse the cell type changes at each defined osteogenic induction stage. Thus, novel and detailed analyses should be applied to evaluate the differentiation process of hPSCs.

As we know, during in vivo embryonic development, the mesoderm and ectoderm cells will differentiate into mesenchymal cells, which can further differentiate into osteogenic precursor cells and osteoblasts by intramembranous or endochondral ossification [7, 8]. This is a process in which the telomerase activity of cells is gradually reduced to zero. Moreover, it has been reported that cell telomerase activity and the cell cycle are highly correlated with cell fate regulation throughout the differentiation process [9, 10]. At the same time, osteogenic differentiation of stem cells is accompanied by the regulation of early osteogenic marker proteins like RUNX2. Therefore, there is great value in performing quantitative measurements for these important indicators, aiming to enhance our understanding of the osteogenic differentiation process of hPSCs.

In this present study, H9 hESCs and hNF-C1 hiPSCs at the Matrigel surface were differentiated into osteoblast-like cells in osteogenic induction medium for 35 days. To clarify the differentiation process, the dynamic expression of markers was monitored by reverse transcription-polymerase chain reaction (RT-PCR) and immunofluorescence. Calcium nodule content and ALP activity were separately determined using alizarin red staining (AS) and ALP staining. Moreover, flow cytometry was used to quantitatively measure the expression of the critical marker protein RUNX2 and the cell cycle in the cell samples. In addition, nuclear staining was used for cell counting. Furthermore, cell telomerase activity was detected as a potential indicator to analyse the cell types at defined time points. Finally, we established a schematic representation for characterizing the change of makers and cell types during the osteogenic differentiation of hPSCs. This study has contributed valuable knowledge about the osteogenic differentiation of monolayer cultured hPSCs, accelerating the development of better in vitro osteogenic differentiation systems.

## Materials and methods

### Materials

Ethylene diamine tetraacetic acid (EDTA), ascorbic acid, sodium glycerophosphate, dexamethasone, cetylpyridinium bromide, alizarin red S and Triton-X100 were obtained from Sigma-Aldrich (USA). Foetal bovine serum (FBS), αMEM medium, non-essential amino acid (NEAA), β-mercaptoethanol, L-glutamax and penicillin/streptavidin were purchased from Gibco (USA). Cell culture plates and Matrigel were bought from Corning (USA). Methanol, absolute ethanol, chloroform, hydrochloric acid and isopropanol were obtained from BCIGC (China). Bovine serum albumin (BSA), phosphate buffer, N-hydroxysulfosuccinimide sodium salt (NHSS) and 1-ethyl-3-(3-dimethylamino propyl) carbodiimide (EDC) were purchased from Aladdin (China). BCIP/NBT alkaline phosphatase colouring kit and ALP quantitative detection kit was acquired from CWBIO (China). SYBR Green I and TRIzol were bought from Takara (Japan). Paraformaldehyde was obtained from Solarbio (China). Quartz crystal microbalance chips were obtained from HRbio (China). Cell counting kit-8 (CCK8) was purchased from Dojindo (Japan). RevertAid™ First Stand cDNA Synthesis Kit was gained from Thermo (USA). Cell cycle assay reagent was obtained from KoradBio (China). E8 medium was acquired from Cellapy (China). DAPI stain was purchased from Roche (Switzerland). Carboxyl functionalized QCM chips were provided by Dongwei BiologicalTechnology Co., LTD (China).

### Cell culture in vitro

H9 hESCs and hNF-C1 hiPSCs were provided as gifts as described previously [11]. Both cell types were cultured on 6-well cell culture plates after coating with Matrigel at a dilution rate of 1:80. The medium used to maintain pluripotency in the experiment was the well-defined E8 medium, and it was changed every day. After growth to approximately 80% confluence, cells were passaged at a split ratio of 1:4 by exposure to 0.5 mM EDTA for 4~5 min at 37 °C.

### Osteogenic differentiation

When grown into 80% confluence, hPSCs on the Matrigel surface were transferred into osteogenic medium (OM) that consisted of αMEM medium, 15% FBS, 1% NEAA, 0.1 mM β-mercaptoethanol, 1% penicillin/streptavidin, 5 μg mL^− 1^ ascorbic acid, 10 mM sodium glycerophosphate and 10^− 8^ M dexamethasone. The OM was changed freshly every 2 days for 35 days. After induction for different times (0 days, 3 days, 7 days, 14 days, 21 days, 28 days and 35 days), the cells were observed using a phase-contrast microscope (CKX31SF, Olympus, Japan) with a CCD camera (MP3.3-RTV, Olympus, Japan), and their viability was detected using the cell counting kit-8 reagent.

### Cell telomerase activity measurement

The telomerase activity of the cell samples throughout the osteogenic differentiation was quantitatively measured using a previously reported method based on a quartz crystal microbalance (QCM) [12]. Briefly, each of 1 million single cells was lysed in 150 μL CHAPS lysis buffer for 30 min on ice. Centrifugation at 12,000 r min^− 1^ and 4 °C for 20 min was performed to extract the supernatant containing telomerase. Subsequently, the protein content was measured using a BCA protein concentration determination kit according to the manufacturer’s instructions [13]. The protein concentration of the sample was adjusted by DPBS with the minimum protein concentration as a reference. To measure the cell telomerase activity, the NHSS/EDC activated QCM chip was immediately incubated with the primers (5′-NH2(CH2)6TTTTTTTTTTAATCCGTCGAGCAGAGTT-3′) and the DNA assembly solution for 3 h. The pre-treated chips were placed into a QCM reactor and then underwent the same processes to detect the frequency changes relating to cell telomerase activity as we previously described [12].

### Quantitative real-time RT-PCR

Cell samples were extracted using TRIzol reagent and total RNA was extracted through the chloroform-isopropanol precipitation method. The total RNA was converted into cDNA using a RevertAidTM First Stand cDNA Synthesis Kit. The mRNA of the samples was detected through quantitative real-time polymerase chain reaction (RT-PCR) using SYBR Green I via an ABI 7500 RT-PCR machine (Applied Biosystems, USA). Three parallel samples were set for each sample, and each replicate was tested in three independent replicates. Quantitatively detected genes contained the internal control gene (*ACTB*), pluripotency marker genes (*OCT-4, NANOG*), osteogenic differentiation-related genes (*RUNX2*, *ALP*, *COL1A1*, *OCN*) and telomerase reverse transcriptase (*TERT*) gene. The primer sequences of these genes are shown in Table S1.

### Immunofluorescence

After osteogenic differentiation for varying times (0 days, 3 days, 7 days, 14 days, 21 days, 28 days, and 35 days), hPSCs in 12-well cell culture plates were fixed in 4% paraformaldehyde for 30 min at room temperature (RT). Fixed samples were used to detect the protein expression of OCT-4, RUNX2, COL1A1, and OCN by immunofluorescence. Briefly, cell samples were permeated for 30 min with 0.2% Triton-X100 and blocked with 3% BSA for 2 h at RT. The samples were, respectively, incubated overnight with primary antibodies at 4 °C. After washing with DPBS 3 times, the cells were incubated with the corresponding fluorescently labelled secondary antibodies in the dark for 1 h. Finally, the samples were stained for 5 min at RT with DAPI that was diluted in DPBS at 1:5000. All stained cell samples were observed and photographed using a confocal fluorescence microscope (Axiovert 200 M; Carl Zeiss Jena, Germany). Meanwhile, the cell numbers were counted using ImageJ software based on the DAPI staining. The primary antibodies and corresponding secondary antibodies are shown in Table S2.

### Flow cytometry study

After incubation for up to 35 days, the hPSCs were digested into single cells and fixed with 1% paraformaldehyde. The cells were permeated for 30 min in 200 μL pre-cooled 90% methanol solution on ice. Subsequently, the sample was washed twice with the flow buffer (DPBS containing 2% FBS) and incubated with mouse anti-human RUNX2 monoclonal antibody at a dilution rate of 1:200 in flow buffer for 30 min at 37 °C. This was followed by incubation with secondary antibodies of Fluor 488-labelled goat anti-mouse IgG at a dilution rate of 1:500 in DPBS. In addition, for cell cycle analysis, single cells were fixed in pre-cooled 75% ethanol at 4 °C for 24 h. Before the flow cytometry study, the cells were incubated in 500 μL cell cycle assay reagent for 30 min at 4 °C in the dark. Finally, all of these cell samples were analysed by a BD FACS Calibur System (BD, USA) and Flowjo software.

### Alkaline phosphatase assay

Cell samples were fixed in absolute ethanol for 30 min, and then stained using a BCIP/NBT alkaline phosphatase colouring kit according to the instructions. After washing with distilled water 3 times, the stained samples were observed with an inverted microscope containing a CCD (Olympus, Japan). In addition, the plates were photographed using a mobile phone. Moreover, the ALP activity of these cell samples was detected using an ALP quantitative detection kit according to the instructions as we previously described [13].

### Determination of calcium nodules content

hPSCs cultured on 12-well cell culture plates were fixed in 4% paraformaldehyde for 30 min. After washing 3 times with DPBS, they were incubated with 500 μL 2% alizarin red (0.01 M Tris buffer, pH = 4.2) for 20 min at room temperature. After repeatedly rinsing with distilled water until the solution was clarified, cells and plates were photographed as previously mentioned. To quantitatively measure the deposited alizarin red S, 500 μL 1% (m/v) cetylpyridinium bromide solution was added into each well of the plate. After the overnight reaction, 100 μL solution from each well was transferred into new 96-well plates and the absorbance at 490 nm was measured using a Bio-Rad full-wavelength microplate reader (Bio-Rad, USA). Three replicate wells were set for each experimental group, and the absorbance value of each well was measured 3 times.

### Statistical analysis

All data were statistically analysed using Student’s *t* test and expressed as the mean ± standard deviation. The difference was considered significant when *p* < 0.05. Three parallel samples were set for each quantitative research, and each parallel was tested in three independent replicates.

## Results

### Analysis of cell morphology and cell viability

**Fig. 1:**
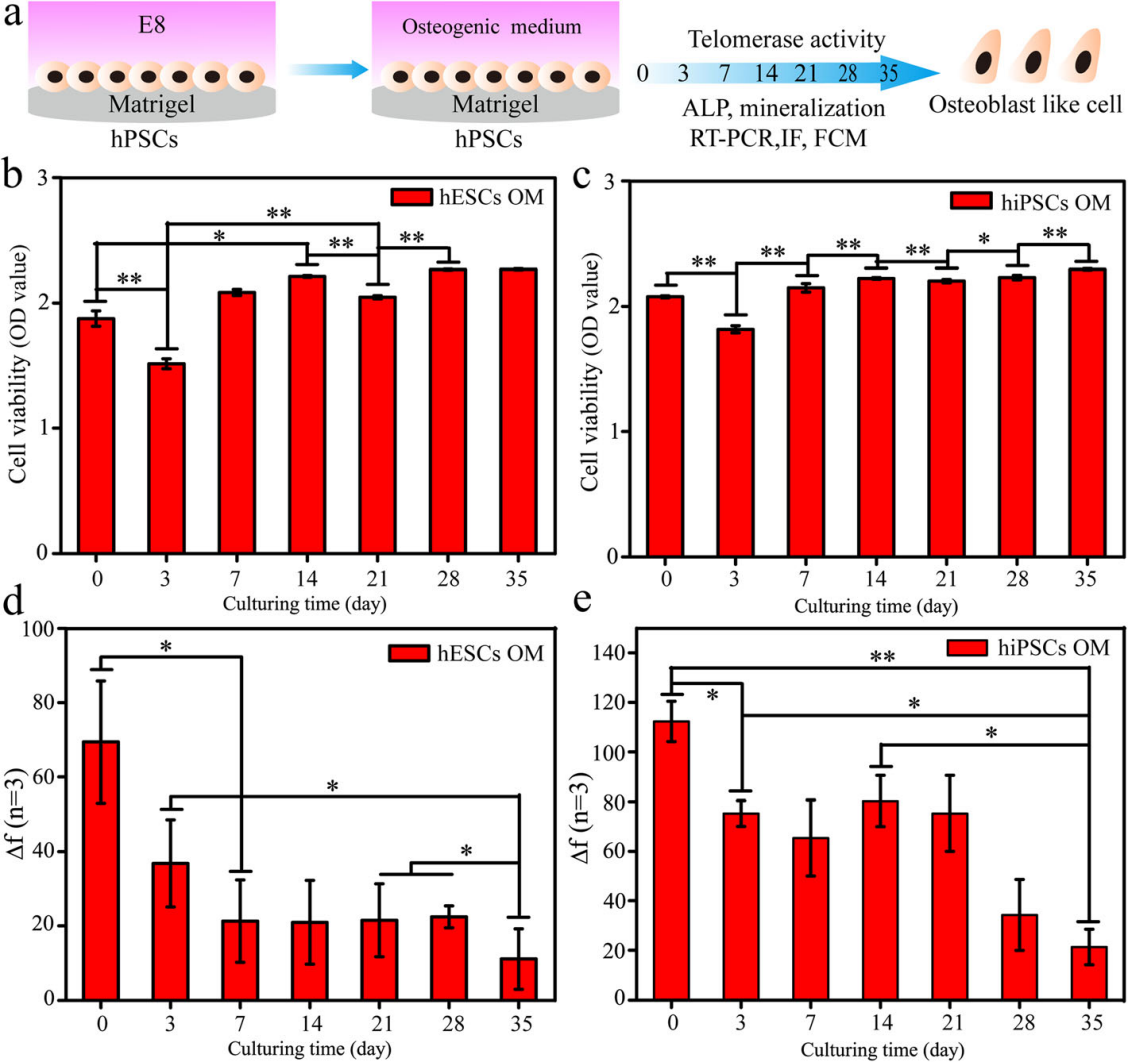
Analyses of cell viability and telomerase activity for hPSCs during 35 days of osteogenic differentiation. **a** A schematic diagram of the experimental protocol. **b**, **c** After osteogenic induction for various days (0, 3, 7, 14, 21, 28 and 35), the cell viability of hESCs (**b**) and hiPSCs (**c**) was detected using the CCK8 reagent. **d**, **e** The telomerase activities of hESCs (**d**) and hiPSCs (**e**) were measured by a quantitative method based on a quartz crystal microbalance (QCM). *Represents *p* < 0.05 (*n* = 3)

For H9 hESCs and hNF-C1 hiPSCs on the Matrigel surface, the E8 medium was changed to the widely used OM containing FBS, ascorbic acid, glycerophosphate and dexamethasone for 35 days after the cells had reached approximately 80% (Fig. 1). Before differentiation, both hESCs and hiPSCs exhibited typical undifferentiated morphologies with a clear clone edge and a high nucleo-cytoplasmic ratio (Fig. S1a-b). After incubation in OM for 3 days, the cell colonies of hPSCs became loose with a large number of dead cells appearing in the medium, resulting in a significantly decreased cell number as that confirmed by CCK8 assay (*p* < 0.01) (Fig. 1b, c). In addition, the cell numbers were obviously increased from this time point throughout 35 days of culture (Fig. 1b, c). Then, many cobblestones or spindle-shaped cells were observed after differentiation for 7 days and 14 days (Fig. S1a-b). With the increasing differentiation time to 35 days, increasing numbers of cells showed irregular cell morphology (Fig. S1).

### Cell telomerase activity was reduced during the osteogenic differentiation of hPSCs

The cell telomerase activity was measured for both hESCs and hiPSCs in each week using a quantitative method based on QCM as we recently reported [11]. In this method, frequency changes (Δ*f*) show a positive correlation with cell telomerase activity.

As shown in Fig. 1d, e, the Δ*f* of the cells decreased with the increase of differentiation time to 7 days, revealing that both hiPSCs and hESCs were differentiated into cells with reduced cell telomerase activity. Surprisingly, consistent cell telomerase activity results were measured for the hESCs over the 7~28 days. Moreover, the telomerase activity of hiPSCs after culturing for 14 days (80 ± 10 HZ) was slightly higher than that of cells within a culture time of 7 days (65 ± 15 HZ) (Fig. 1e).

### Cell cycle changes during the osteogenic differentiation of hPSCs

**Fig. 2:**
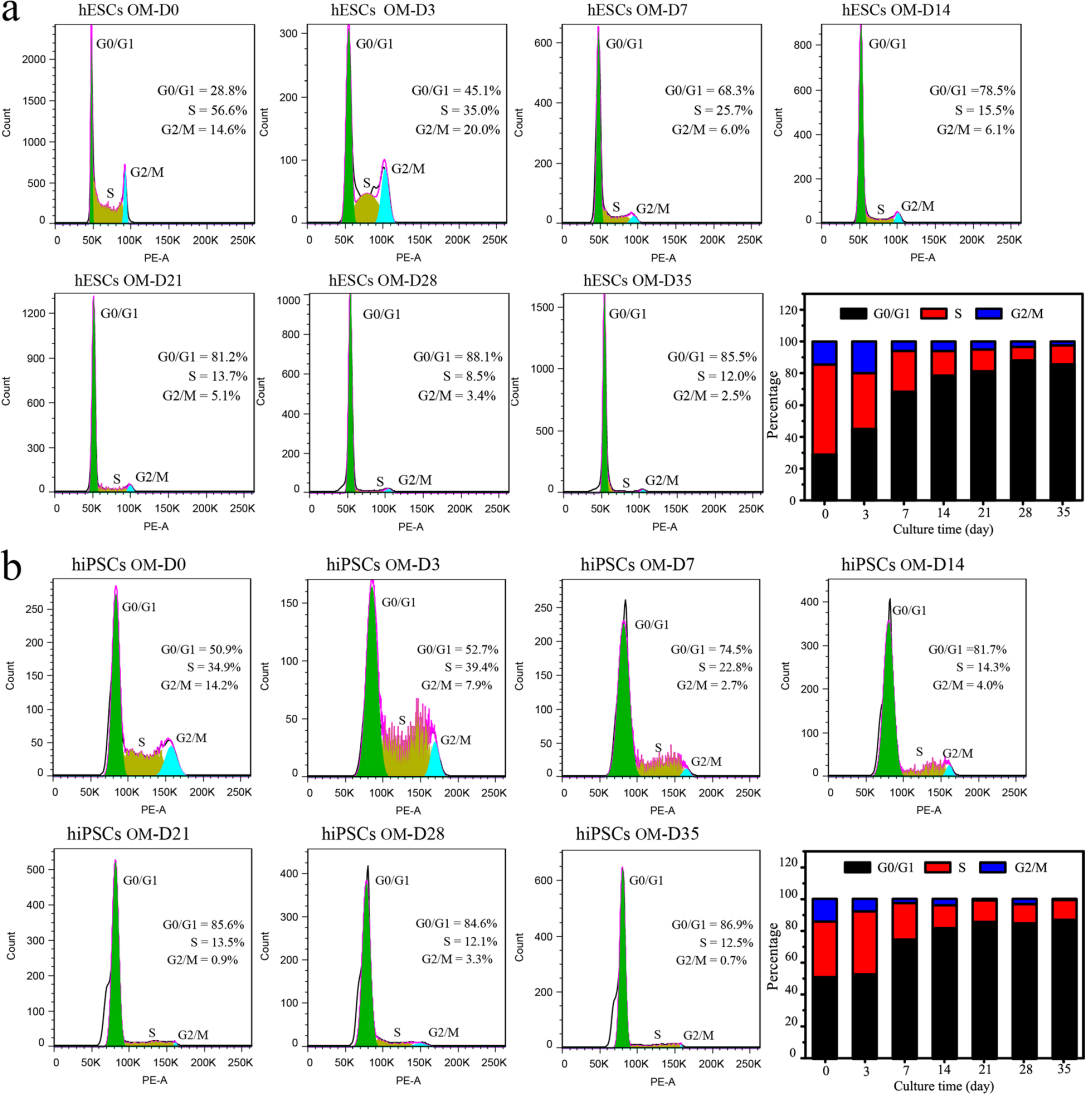
Analyses of the cell cycle for hPSCs during 35 days of osteogenic differentiation. **a**, **b** The cell cycle changes of the hESCs (**a**) and hiPSCs (**b**) after induction for different times (0 days, 3 days, 7 days, 14 days, 21 days, 28 days and 35 days) were studied using flow cytometry

In this study, a cell cycle detection reagent and flow cytometry were applied to investigate the cell cycle changes in hPSCs during 35 days of osteogenic differentiation. hPSC incubation in the induction medium activates the developmental process, resulting in a reshape cell cycle with a prolonged G1 phase and whole cell division time [14]. Although both cells were grown to approximately 80% confluence before differentiation, the percent of cells in the S phase stage for the hESCs (56.6%) was higher than that of the hiPSCs (34.9%), suggesting hESCs harbour better proliferation ability than hiPSCs (Fig. 2). However, similar results were found for cells at the G2/M phase stage. Then, the percent of cells in the G2/M and S phase stage for both hESCs and hiPSCs decreased with the augment of induction time to 35 days, resulting in more cells in the G0/G1 phase stage. Many hPSCs remained in the S/G2/M phase stage after 3 days of culture, which could be the reason why the viability of the cells was increased from day 3 to day 7. Moreover, a decreased proliferation rate was combined with the medium’s selective killing effect, which can explain previous results showing that only a slightly higher cell viability was detected during 35 days of differentiation (Fig. 2a).

### Expression of gene and protein markers in the induced hPSCs

The differentiation of stem cells shows the dynamic changes in the expression of related gene/protein markers at each stage [7]. In this study, after osteogenic differentiation for varying times (3, 7, 14, 21, 28 and 35 days), we analysed the expression of the pluripotent genes (*OCT-4* and *NANOG*), telomerase reverse transcriptase (*TERT*) and osteogenesis-related genes (*RUNX2*, *ALP*, *COL1A1* and *OCN*) in hESCs and hiPSCs. At the same time, immunofluorescence was used to detect the protein expression of OCT-4, RUNX2, COL1A1 and OCN in these cell samples. In addition, for the critical marker the RUNX2 protein, its expression in hPSCs was further quantitatively measured using flow cytometry.

**Fig. 3:**
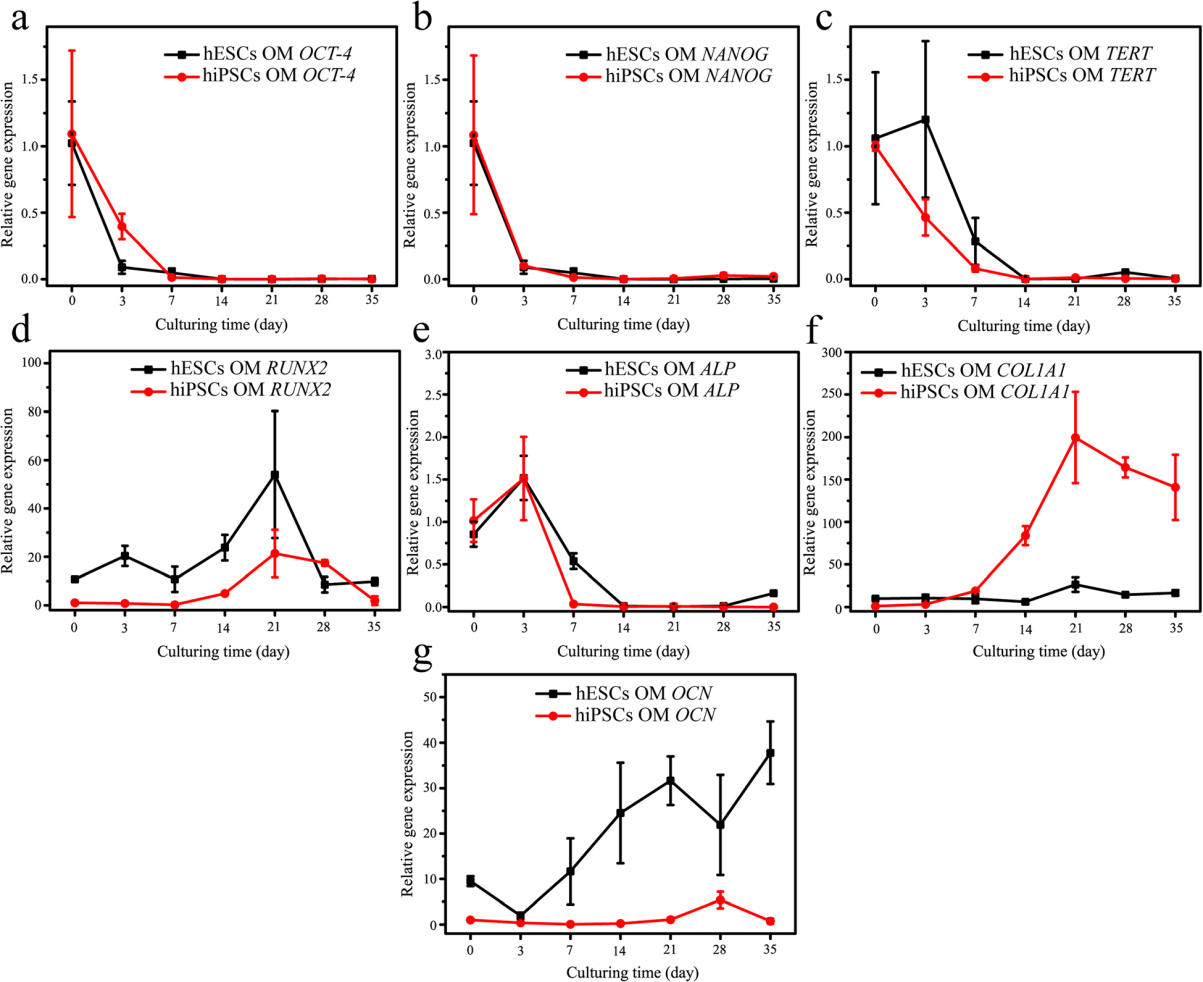
The expression of marker genes in hPSCs during osteogenic differentiation. **a–g** After osteogenic induction for up to 35 days, the expression of marker genes such as *OCT-4* (**a**), *NANOG* (**b**), *TERT* (**c**), *ALP* (**d**), *RUNX2* (**e**), *COL1A1* (**f**) and *OCN* (**g**) in the cell samples was measured by RT-PCR. *n* = 3

**Fig. 4:**
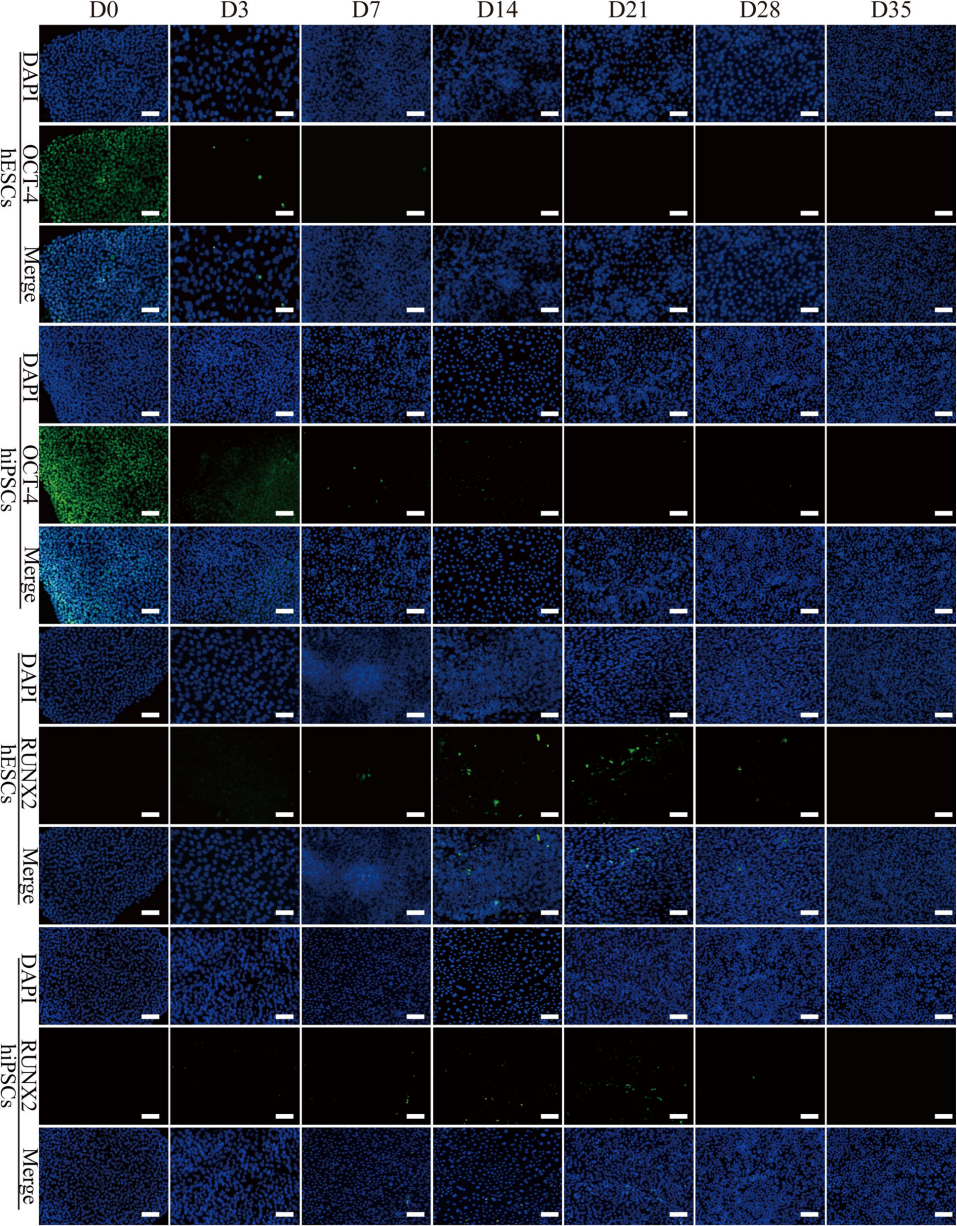
The expression of OCT-4 and RUNX2 in hPSC samples during osteogenic differentiation. The expression of OCT-4 (green) and RUNX2 (green) in the H9 hESCs and hNF-C1 hiPSCs after osteogenic induction for the indicated days were detected by immunofluorescence. The nucleus is shown in blue by DAPI staining. Scale bars, 100 μm

As shown in Fig. 3, the expression of *OCT-4* and *NANOG* in cells decreased rapidly after the replacement of OM for 3 days (*p* < 0.05) (Fig. 3a–c). Surprisingly, repeated experiments found that the gene expression of *TERT* in hESCs was not reduced at that time point, which may be because these initially differentiated cells retained high self-renewal ability. Then, the *OCT-4* and *NANOG* genes virtual were not expressed after 7 days of osteogenic differentiation, and the *TERT* gene was barely expressed after 14 days of culture (Fig. 3a–c). Consistently, immunofluorescence detection showed similar results of OCT-4 expression. Both cell types positively expressed OCT-4 before differentiation (Fig. 4). Then, the number of positive expression cells was remarkably decreased after being transferred into the induction medium, and they had almost completely disappeared after 14 days of culturing. Consistent with previous reports, these results confirmed that the osteogenic differentiation of hPSCs is a process of pluripotency reduction [15].

**Fig. 5:**
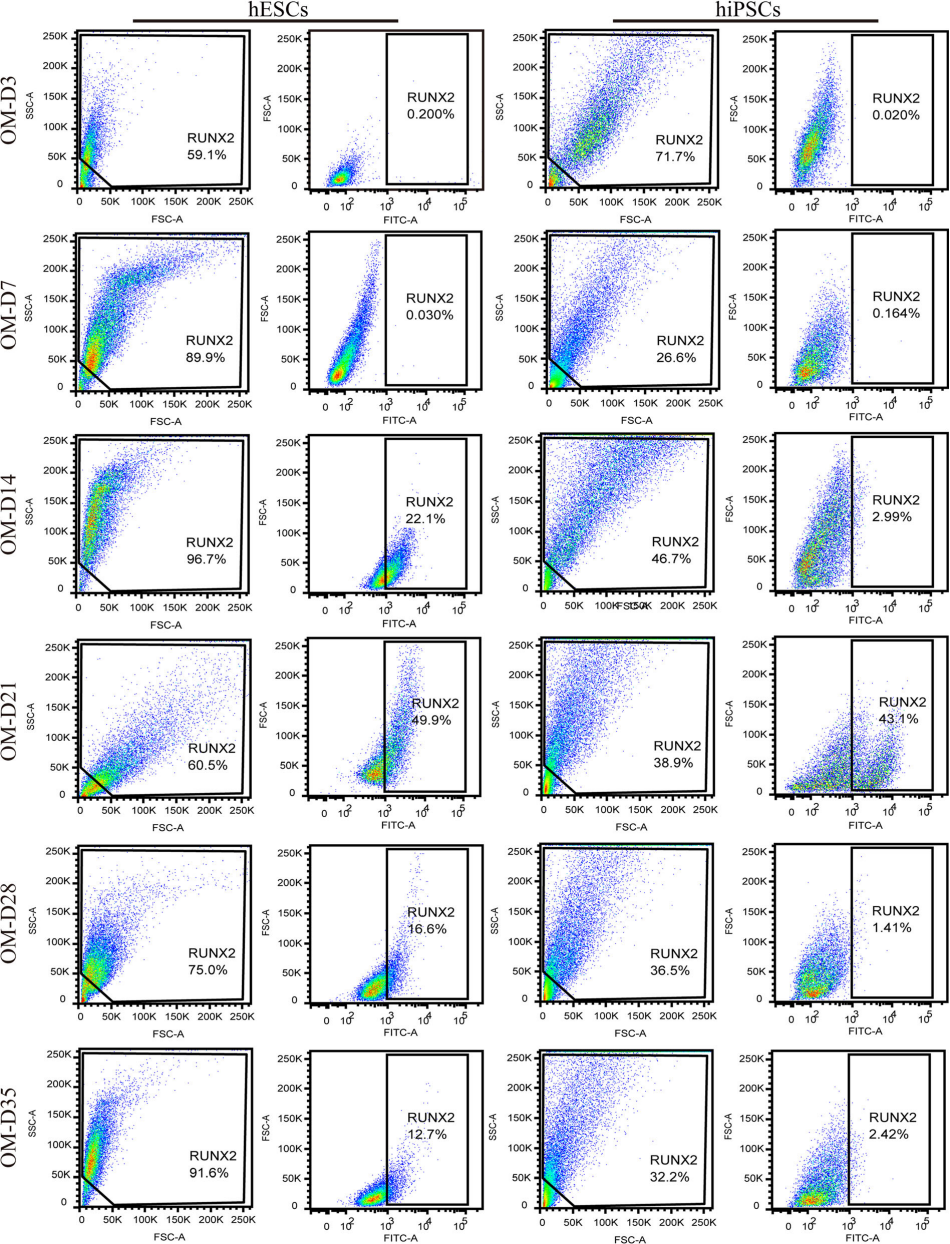
The measurements for RUNX2-positive expression in hPSCs during osteogenic differentiation. After osteogenic induction for different days (0, 3, 7, 14, 21, 28 and 35), the expression of RUNX2 in the cells was measured by flow cytometry, and undifferentiated hPSCs were used as the control

For the osteogenic markers, *RUNX2* is a significant multifunctional transcription factor during the osteogenic differentiation of stem cells, and it can regulate the transcription of other osteoblast-related genes such as *COL1A1* and *OCN* [16, 17]. The analysis of RT-PCR showed that the expression of the *RUNX2* gene in hPSCs began to rise steadily after 7 days of culture, and it reached peak values at 21 days (Fig. 3d). Then, several cells positively expressing RUNX2 were found in both hESCs and hiPSCs after 14 days and 21 days of induction as confirmed by immunofluorescence (Fig. 4). Moreover, as shown in Figure S2, we counted the number of DAPI-stained cells and RUNX2-positive expressed cells in the immunofluorescent images using ImageJ software, aiming to acquire semi-quantitative data showing the fraction of RUNX2-positive cells in the whole population of cells. It is found that the mean values for the percent of RUNX2-positive cells was only about 1~3% for hESCs and 2~7% for hiPSCs throughout the 35 days of induction. Compared to the immunofluorescence results, although a consistent tendency was found for the flow cytometry results as shown in Fig. 5, the expression level was quite different between them. Specifically, the expression of RUNX2 protein in both cell types was increased with the augment of culture time to 21 days and reached peak values of 49.9% and 43.1% for the hESCs and hiPSCs respectively (Fig. S3). Apparently, a much higher expression level was detected using flow cytometry technology in comparison to immunofluorescence analyses (Fig. S2 and Fig. S3), which may be due to very differentiated cells expressing limited amounts of RUNX2 protein and flow cytometry assays having better sensitivity. In addition, after induction times for 14 days, 28 days and 35 days, 12.7~22.1% hESCs positively expressed RUNX2. However, except for the time point of 21 days, nearly negative results were detected for hiPSCs. These results proved that the flow cytometry assay is a very important quantitative analysis to investigate the osteogenic induction of hPSCs, and the differences in cell lines and cell states would affect the expression of RUNX2.

Then, the expression of other osteogenic differentiation markers ALP, COL1A1 and OCN was also analysed by RT-PCR and immunofluorescence. ALP is one of the alkaline phosphatase isozymes that is ubiquitously expressed in bone-forming cells, and it plays a critical role in early osteogenesis and hydrolyses various types of phosphates to promote cell maturation and calcification [18]. Thus, ALP is considered to be an early osteogenic differentiation marker. For both hESCs and hiPSCs, the expression of the *ALP* gene peaked after 3 days of induction, and then rapidly decreased to a quite low expression level from the 14th day onward (*p* < 0.05) (Fig. 3e). These results may suggest that hPSCs undergo an early differentiation process towards osteoblasts during 3~7 days.

**Fig. 6:**
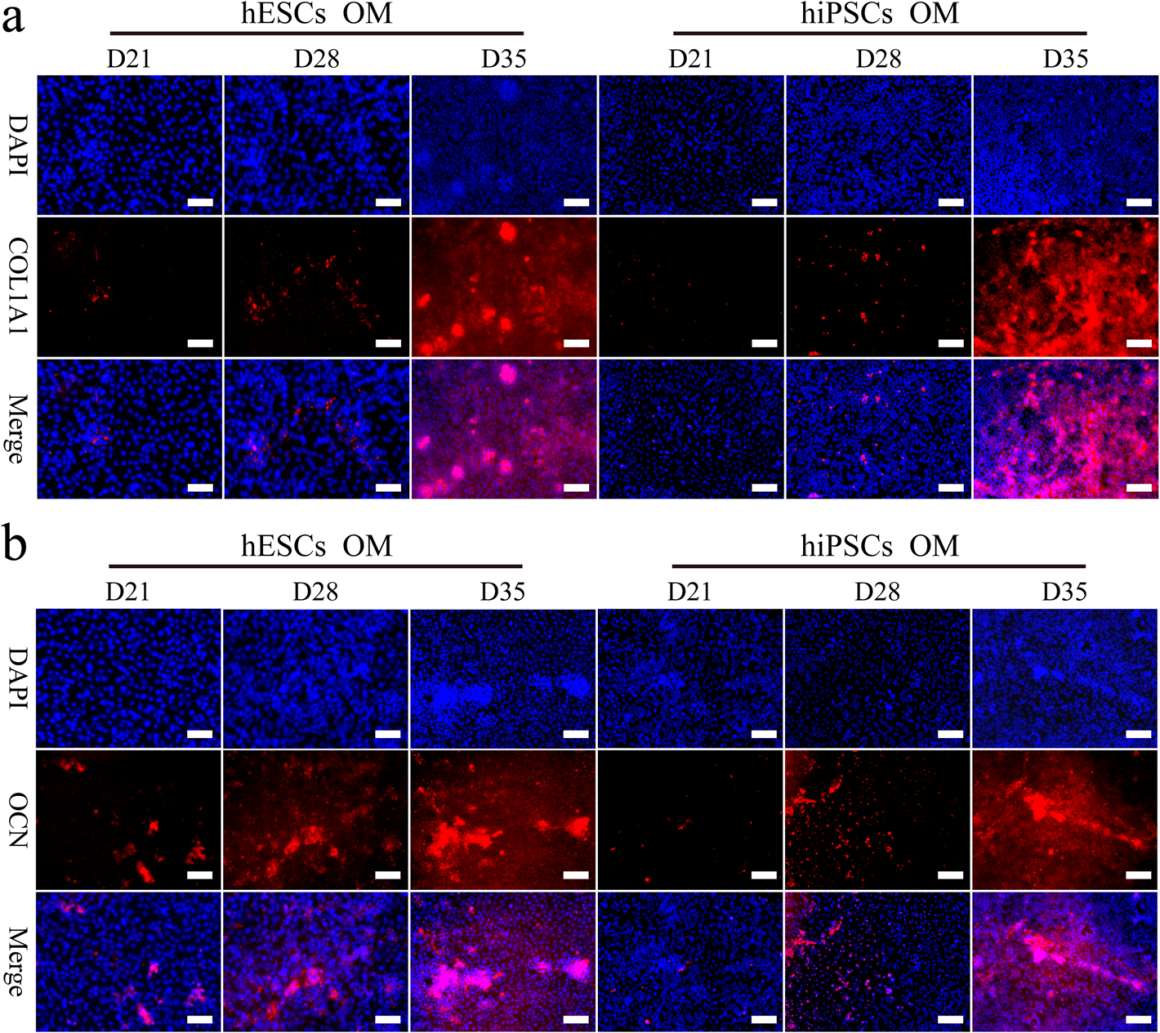
The expression of COL1A1 and OCN in the induced hESCs and hiPSCs. After osteogenic induction for 21 days, 28 days or 35 days, the expression of COL1A1 (red) and OCN (red) in the cell samples were detected by immunofluorescence. The nucleus is blue from DAPI staining. Scale bars,100 μm

As shown in Fig. 3f, the late osteogenic differentiation marker gene *COL1A1* in the hPSCs was upregulated from day 14, peaked at day 21, and then was downregulated through 35 days. These results are similar to previously reported studies [19, 20]. To our surprise, although the expression trend of the two cell lines was almost consistent, the expression of the *COL1A1* gene in hiPSCs with a differentiation time more than 14 days was much higher than that in hESCs (Fig. 3f). Similarly, significant differences in the gene expression of *OCN*, a marker of osteoblast formation, were found between the two cell lines. For the hESCs, after a slight decrease during the initial 3 days, the expression of the *OCN* gene was increased with the augmentation of culture time to 35 days except for the time point of day 21 (Fig. 3g). Interestingly, hiPSCs expressed the *OCN* gene at a low level after induction, and its upregulation was found until day 21. In addition, the expression of COL1A1 and OCN protein in these cell samples was detected during the late stage of osteogenic induction (21 days, 28 days and 35 days) using immunofluorescence techniques (Fig. 6). We found that the expression of both proteins in the hPSCs was gradually increased from 21 to 35 days. It has been reported that an apparent downregulation of *OCN* is associated with the accumulation of low levels of hydroxyapatite in the later stages [21]. In addition, previous studies reported that OCN inhibits mineralization but is highly expressed at the end of the maturation of the extracellular matrix and undergoes rapid downregulation before mineralization, then gradually increases [22–24]. Therefore, these results may suggest that hPSCs form mature extracellular matrix during the culturing period of 21~28 days. In summary, our results preliminary indicated that hESCs and hiPSCs undergo similar expression changes for markers relating to pluripotency and osteogenic differentiation, but not for extracellular matrix protein markers.

### ALP and alizarin red staining analysis

ALP staining is commonly applied to identify reprogrammed hPSCs and its osteogenic differentiation process. Both hESCs and hiPSCs expressed ALP at a high level before differentiation (Fig. S5). After culturing in induction medium for 3 days, many stained cells were found in the hiPSCs, but not for the hESCs. Then, the ALP expression in both cells decreased rapidly and almost disappeared at day 14. With the prolongation of osteogenic differentiation time, the ALP activity of the cells switched to increasing until 28 days of culture (Fig. S6). This trend is similar to the results of previous studies [13, 25].

**Fig. 7:**
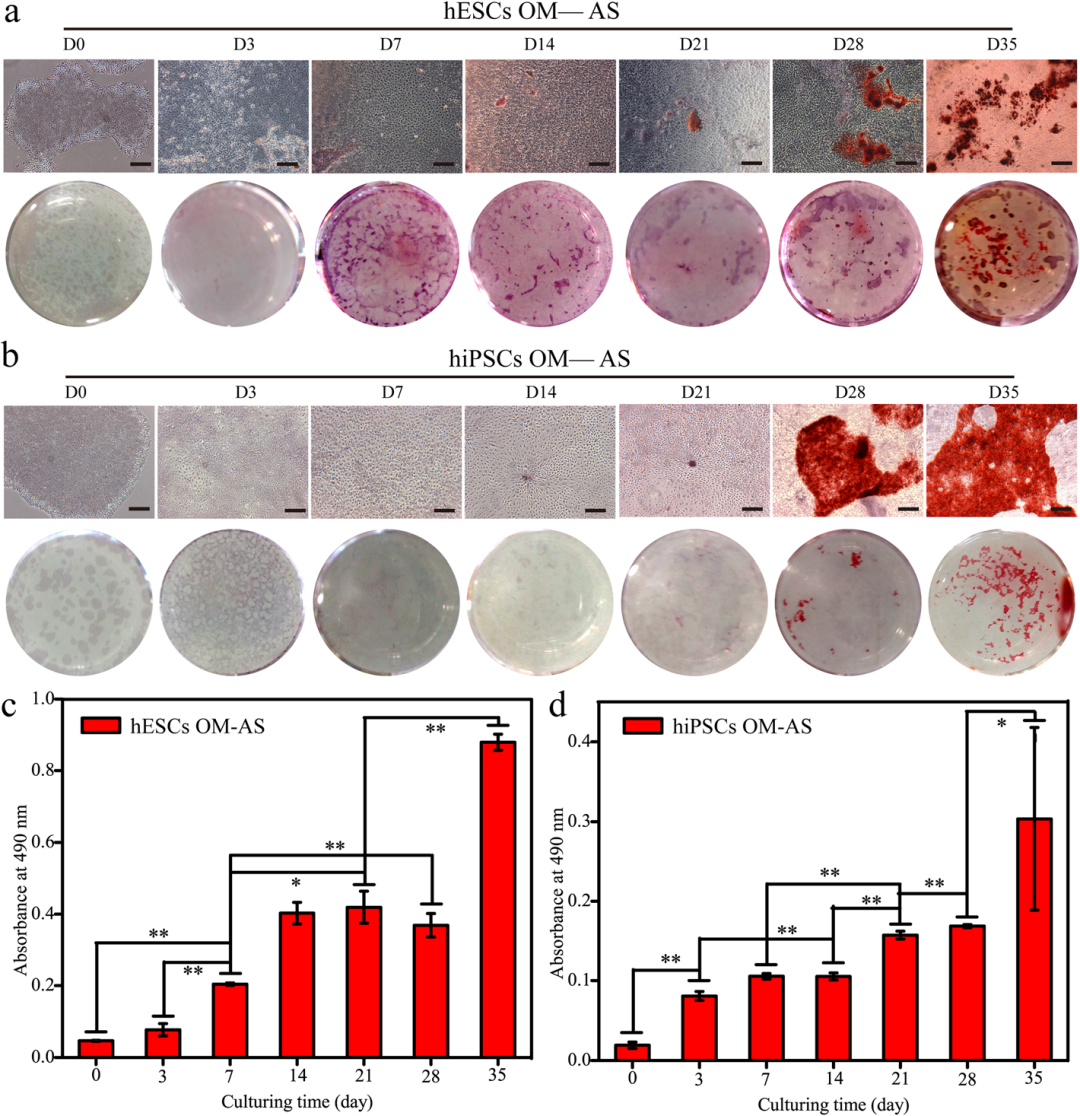
The Alizarin red staining analyses for the hPSCs during osteogenic differentiation. **a**, **b** Cell morphology and culture plate photograph of alizarin red staining of hESCs (**a**) and hiPSCs (**b**) after culturing in induction medium for up to 35 days. Scale bars, 200 μm. **c**, **d** Cetylpyridinium bromide solution was applied to dissolve the deposited alizarin red and the absorbance at 490 nm was measured. *Represents *p* < 0.05 (*n* = 3)

In addition, AS was applied to study the calcium-containing nodule formation (Fig. 7). As shown by both qualitative and quantitative results, more deposited alizarin red was detected with the increase of culture time to 35 days, especially at the time points of 28~35 days. Typical calcium nodules were found after induction for 14 days for hESCs, but the time point was 28 days for the hiPSCs (Fig. 7a, b). This is the reason why the quantitative results showed that the calcium salt deposition of hESCs was approximately 2 times higher than that of hiPSCs during the osteogenic differentiation at 35 days (Fig. 7c, d). Interestingly, we observed a slight downregulation of calcium nodules in the hESCs after induction for 28 days (*p* > 0.05). All of these findings were consistent with the *OCN* gene expression results as confirmed by RT-PCR, which may be because the expression level of OCN is closely associated with both the production and maturation of mineral species in cells [26]. The results of RT-PCR and AS staining demonstrated that H9 hESCs harbour much better performance than hiPSCs in extracellular matrix synthesis, and this difference should be considered when evaluating the osteogenic differentiation among research using different hPSCs cell lines.

## Discussion

In this study, monolayer cultured hESCs and hiPSCs were induced into osteoblast-like cells using an induction medium containing FBS and osteogenic differentiation factors. Due to a relatively high cell density of 80% before differentiation, similar cell viability results with no apparent cell morphology changes were found for both cells during the differentiation process over 35 days (Fig. 1b, c). Notably, the values of cell viability assay were reduced at day 3 for both cells. It is well known that the epithelial-to-mesenchymal transition (EMT) is a critical step for mesoderm differentiation of hPSCs. During this process, epithelial cells lose their membrane junctions and apical-basal polarity, reorganize their cytoskeleton, undergo a change in the signaling pathways that define cell shape and reprogram gene expression. The epithelial cell suffers several changes involving transcription factor activation, specific cell-surface protein expression, cytoskeletal protein reorganization and expression, ECM-degrading enzyme production and microRNA expression [27]. Notably, these changes could cause cell apoptosis [28]. Thus, apart from factors that include high initial cell density, intercellular contact inhibition and accumulation of metabolites, cell apoptosis should play an important role in the reduction of cell viability after osteogenic induction for 3 days.

Cell telomerase activity plays a key role in the self-renewal of cells, and it is gradually downregulated during the in vivo embryonic development [9]. Germ cells have high telomerase activity, while it disappears when they change into terminally differentiated cells [12]. Consistently, we have confirmed that the telomerase activities of hPSCs, human bone marrow mesenchymal stem cells (hBMSCs) and MG63 osteoblasts decreased successively [12]. Therefore, cell telomerase activity can be applied as one of the important quantitative markers to monitor the in vitro osteogenic differentiation process of hPSCs. Our results showed that the telomerase activity of hPSCs reduced at the first week, but unexpected results were found for both cells during the following induction (Fig. 1d, e). It may be because heterogeneous cells with low differentiation efficiency were obtained throughout the osteogenic differentiation process.

To our knowledge, the growth and development of hPSCs are highly correlated with their cell cycle characteristics [10]. Cell fate switches are always accompanied by the changes in the cell cycle. It has been reported that a shortened G1 cell cycle shows benefits for the self-renewal of hESCs [29, 30]. Therefore, cell cycle analyses are important to exhibit significance to determine the osteogenic differentiation progress of hPSCs, which has rarely been researched before. Our cell cycle analysis indicated that more cells would be arrested in the G0/G1 phase with the increase of osteogenic differentiation time to 35 days. During cell development, chromosomes are replicated during the S phase and then segregated to daughter cells during the M phase for cell proliferation, and an exit from the cell cycle at G1 phase is commonly required for terminal differentiation of cells [29, 31]. Notably, the trend of diminishing in the proportion of S phase cells during the osteogenic induction of hPSCs was also consistent with the results of decreasing cell telomerase activity (Fig. 1d). It has been reported that cell telomerase activity highly relevant to cell cycle regulation, and the highest levels of cell telomerase activity occur in the S phase [32, 33]. In fact, in vivo bone development is a process in which the pluripotency and proliferative ability of cells decrease gradually [34]. Similarly, the performances of in vitro self-renewal for hPSCs, human mesenchymal stem cells, osteoblasts and osteocytes are in precipitous decline. Therefore, we think the assay of cell telomerase activity and cell cycle play essential roles in understanding the osteogenic differentiation process of hPSCs.

The osteogenic differentiation of hPSCs is a process in which the expression of markers related to pluripotency and osteogenesis are dynamically changed [24]. It is reported that the mesoderm and ectoderm cells that are derived from hPSCs are the primary source of MSCs, which can further differentiate into pre-osteoblasts and osteoblasts [7, 35, 36]. In this process, RUNX2 expressing pre-osteoblasts will change into cells expressing osterix, ALP and COL1A1 [37]. In addition, mature osteoblasts synthesize a variety of extracellular matrix proteins such as OCN, BSP and OPN, and the positive expression of OCN is generally regarded as an important marker for osteoblasts [37]. Analyses include RT-PCR, immunofluorescence and flow cytometry which were applied to monitor the changes of maker genes or proteins during the differentiation at each week. During the whole 35 days of osteogenic differentiation, similar expression trends were found for most pluripotency and osteogenesis-related markers between hESCs and hiPSCs. It is worth mentioning that we overcame the difficulties of cell numbers and cell dissociation after induction for more than 14 days, and succeeded in obtaining enough cells for the flow cytometry assay. The flow cytometry assay plays a very important role in the quantitative evaluation protein expression and differentiation efficiency.

As proven previously, apparently heterogeneously differentiated cells were obtained throughout 35 days of induction, which is the reason why the CCK8 assay cannot accurately reflect the cell numbers. Moreover, dissociating differentiated cells into single cells using trypsin is a quite difficult process with a low survival rate. Therefore, DAPI staining was applied to enhance the knowledge about the number of cells after culturing for varying days (Fig. S4). When the culturing time was more than 7 days, quite different cell number results were detected between DAPI staining and CCK8 assays. The number of hPSCs was decreased after culturing for 14 days, but similar cell viability results were measured. This is possible because of their increased cell size and cellular metabolic level changes. In addition, analyses of cell telomerase activity and cell cycle proved that cells at this stage harbour a good cell division ability (Fig. 1d, e and Fig. 3a, b). We could conclude that many cells died during this period due to the selective killing effect of OM. Then, the number of hESCs reduced after 21 days of induction, but contrasting results were found for hiPSCs (Fig. S4). This is consistent with previous results showing that hiPSCs at day 14 harbour much higher cell telomerase activity than hESCs (Fig. 1d, e). Finally, the number of hPSCs was increased with the augment of the induction time to 35 days, suggesting very few cells died since the cells have limited proliferation ability during this period as confirmed by cell telomerase activity and cell cycle studies. These results proved that nuclear staining has value in analysing the cell number changes as well as the killing effect of induction medium during the osteogenic differentiation of hPSCs.

**Fig. 8:**
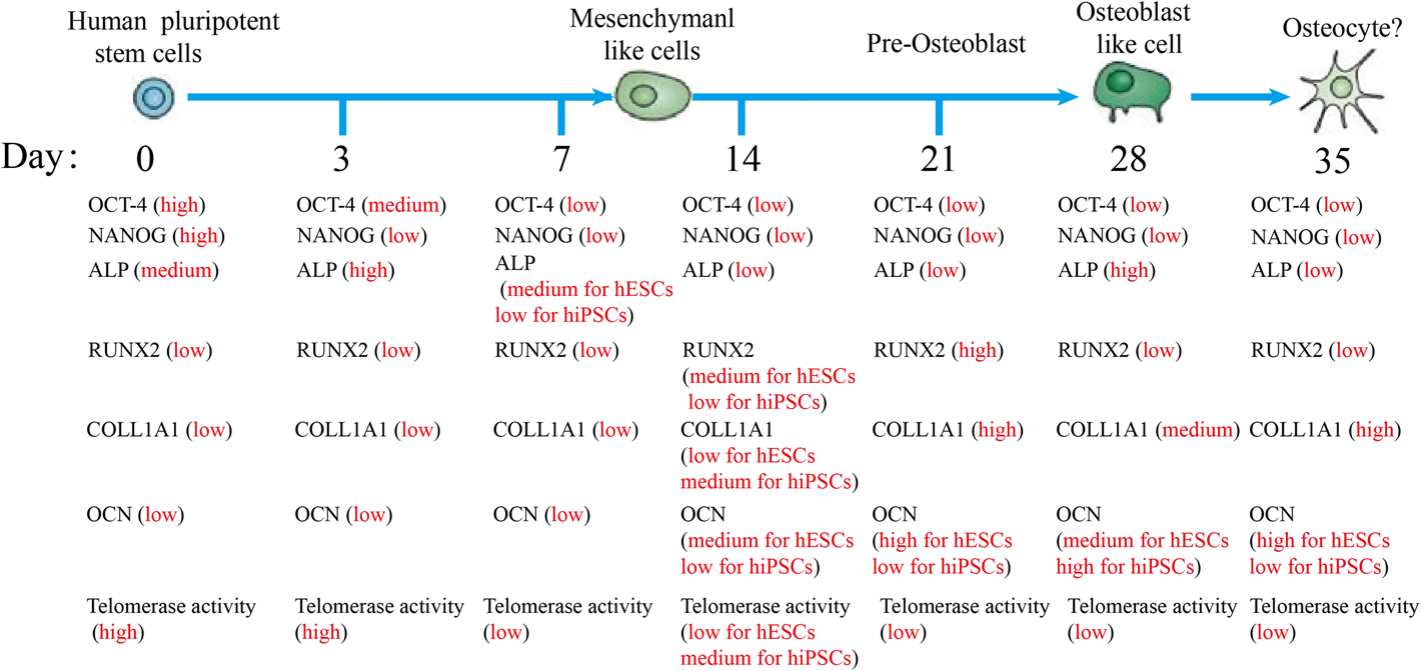
A dynamic map of the osteogenic differentiation of the hPSCs. Expression changes of OCT-4, NANOG, TERT, ALP, RUNX2, COL1A1 and OCN and cell telomerase activity were investigated in the hPSCs during 35 days of osteogenic differentiation. The panels represent (from left to right) hPSCs that were induced for 0 days, 3 days, 7 days, 14 days, 21 days, 28 days or 35 days, which cover the various stages of osteoblastic lineage development

Finally, based on these knowledges, we preliminarily drew a dynamic map for the expression of marker genes and proteins during the osteogenic differentiation of hPSCs (Fig. 8). The expression of pluripotent markers of OCT-4, NANOG and TERT in the cells decreased to a quite low level after osteogenic differentiation for 7 days (Fig. 3a–c). At the same time, the cell telomerase activity and the number of cells in S stage were both at moderate levels (Fig. 1d, e). It has been reported that monolayer cultured hPSCs could be differentiated into MSCs using MSC culture medium [38]. Moreover, the cells are negative for expressing osteogenic markers (Fig. 3d–g). Therefore, we speculated mesenchymal-like cells were obtained at day 7. After induction for 14 days, the cells started to express the osteogenic markers of RUNX2, OCN and COL1A1, which suggested that the MSCs had been differentiated into osteoblast-like cells (Fig. 3d, f, g). Moreover, their expression levels were increased as the osteogenic induction continued (Fig. 3). At the same time, cell cycle analysis indicated that more cells were arrested at the G0/G1 phase with the augment of the differentiation time to 35 days (Fig. 2). Consistently, the telomerase activity of the cells was reduced after 14 days of induction (Fig. 1d, e). Moreover, typical calcium nodules were found in the cell samples after induction for 21 days, and a large amount of AS staining area was found on day 35. According to these results, we speculated that pre-osteoblast-like cells were obtained during 14~21 days of differentiation, and then osteoblast-like cells were induced during 28~35 days.

In a similar research focusing on the time course and gene expression in hESCs during the 25 days of osteogenic differentiation [24]. The authors found that another important osteoblast-specific transcription factor of OSX was peaked at day 15 and decreased to low levels at day 17. OSX plays a role downstream of RUNX2 and both them expressed in pre-osteoblast [39]. This result indicated that the formation of pre-osteoblasts occurred 14 days ago, which is inconsistent to our study. For the reasons, a short embryoid body suspension process was applied to realize cell attachment on the gelatine surface in their studies. Besides, the difference in cell density is supposed to be another reason because the replicative capabilities of pre-osteoblast exceedingly dependent on cell density [40]. It is reported that RUNX2-positive expressing pre-osteoblast cells will differentiate into mature osteoblasts that express the late osteogenic differentiation marker genes [41]. In addition, *COL1A1* in the hPSCs was upregulated from day 14, peaked at day 21, and then was downregulated through 35 days as observed in Fig. 3f. These results are similar to previously reported studies [19, 20]. Moreover, previous studies reported that OCN inhibits mineralization but express at a high level at the end of extracellular matrix maturation. Then, it undergoes rapid downregulation before mineralization, and a gradually increase is followed [22–24]. Separately, Karp et al. found that the expression of OCN was reached a plateau at day 20 and Elerin Karner et al. only took 19 days [21, 24]. Compared with our methods, their cell densities before osteogenic differentiation were lower. In a word, the applications of FBS containing induction medium and quite different differentiation protocols result in the difficulties in the result comparison among published reports.

Although hPSCs have been successfully differentiated into osteoblast-like cells, not good differentiation efficiency was obtained as confirmed by AS staining. It is urgent to further optimize the differentiation process to improve the efficiency, contributing to the establishment of directed induction systems. Moreover, our results showed that the differentiation of hPSCs in that medium typically results in heterogeneous cellular populations, and even the presence of only a small fraction of osteoblasts can yield positive results. For the translation of hESCs and hiPSCs, it could be a very critical step to selectively enrich for osteoblasts within these heterogeneous cell populations. Unfortunately, the commonly applied long-term culture or serially passaging methods are nearly useless, because osteoblasts are terminally differentiated cells with a limited ability of proliferation. More importantly, FBS supports the survival of other type cells too. Thus, we think the establishment of hPSC lines expressing fluorescence-labelled protein makers such as RUNX2, ALP and OCN by using gene editing technologies could be a good alternative method.

This present study has enhanced the understanding of the osteogenic differentiation process of hPSCs, but an accurate definition of various intermediate cells is still a big problem because of a remarkably heterogeneous population of differentiated cells. Subsequently, more specific expression markers should be applied, and primary MSCs and osteoblasts that are extracted from humans could be used as controls. More importantly, we have started an effort to develop a chemically defined in vitro induction system for the stepwise osteogenic differentiation of hPSCs.

## Conclusions

In this study, the osteogenic differentiation process of monolayer cultured hESCs and hiPSCs were analysed in detail. The expression of pluripotency makers was reduced, and dynamic changes with the extension of the differentiation time were found for the osteogenic-related markers. Moreover, it was confirmed that cell telomerase activity, cell cycle, quantitative protein expression of RUNX2 and nucleus staining could be used as valuable evidence to track the cell differentiation processes. Although the hPSCs were successfully induced into osteoblast-like cells in traditional serum-containing osteogenic medium, low expression level of osteogenic-related markers and few calcium nodules were detected throughout the 35 days of induction. This low differentiation efficiency is mainly because a remarkably heterogenous population of differentiated cells was obtained using a too-simple induction method. Therefore, the osteogenic differentiation medium of hPSCs should be optimized by supplementing it with functional compounds at defined stages in a future study. Our study has achieved better understanding of the osteogenic differentiation process of hPSCs, which has value to both optimize the differentiation system and obtain the target mesenchymal-like cells and osteoblast-like cells.

## Supplementary Information

**Additional file 1.** Additional file of supporting information

## Data Availability

The data and materials used and/or analysed during the current study are not publicly available but available from the corresponding author on reasonable request.

## Abbreviations

hPSCs: Human pluripotent stem cells
hESCs: Human embryonic stem cells
hiPSCs: Human-induced pluripotent stem cells
RT-PCR: Reverse transcription-polymerase chain reaction
MSCs: Mesenchymal stem cells
FBS: Foetal bovine serum
ALP: Alkaline phosphatase
RUNX2: Runt-related transcription factor 2
OSX: Osterix
COL1A1: Type I collagen
OCN: Osteocalcin
BSP: Bone sialoprotein
OPN: Osteopontin
TERT: Telomerase reverse transcriptase
EDTA: Ethylene diamine tetraacetic acid
NEAA: Non-essential amino acid
BSA: Bovine serum albumin
NHSS: N-hydroxysulfosuccinimide sodium salt
EDC: 1-Ethyl-3-(3-dimethylamino propyl)carbodiimide
CCK8: Cell counting kit-8
OM: Osteogenic medium
QCM: Quartz crystal microbalance
RT: Room temperature
EMT: Epithelial-to-mesenchymal transition
hBMSCs: Human bone marrow mesenchymal stem cells
AS: Alizarin red staining
