# Supplementary material for: Establishing a deeper understanding of the osteogenic differentiation of monolayer cultured human pluripotent stem cells using novel and detailed analyses
Source: Stem Cell Res Ther. 2021 Jan 7;12:41. doi: 10.1186/s13287-020-02085-9 (PMC7792045; doi:10.1186/s13287-020-02085-9)
Supplement: Supplementary file 1 — Additional file 1. Additional file of supporting information [file 13287_2020_2085_MOESM1_ESM.docx]

**Supporting information for**

**Establishing a deeper understanding of the osteogenic differentiation of monolayer cultured human pluripotent stem cells using novel and detailed analyses**

Ping Zhou^1, a^, Jia-Min Shi^1, b^, Jing-E Song^a^, Yu Han^a^, Hong-Jiao Li^a^, Ya-Meng Song^a^, Fang Feng^a^, Jian-Lin Wang^b^, Rui Zhang^a, b,^ *, Feng Lan^c,^ *

^a^ School and Hospital of Stomatology, Lanzhou University, No.222 Tianshui South Road, Chengguan District, Lanzhou 730000, Gansu Province, PR China.

^b^ College of Life Sciences, Lanzhou University, No.222 Tianshui South Road, Chengguan District, Lanzhou 730000, Gansu Province, PR China.

^c^ National Center for Cardiovascular Diseases, Fuwai Hospital, Chinese Academy of Medical Sciences and Peking Union Medical College, Beijing 100037, PR China.

**Corresponding author**

*Address correspondence to Prof. Feng Lan (e-mail: [fenglan@ccmu.edu.cn](mailto:fenglan@ccmu.edu.cn)) or to Rui Zhang (e-mail: [zhangrui@lzu.edu.cn](mailto:zhangrui@lzu.edu.cn)).


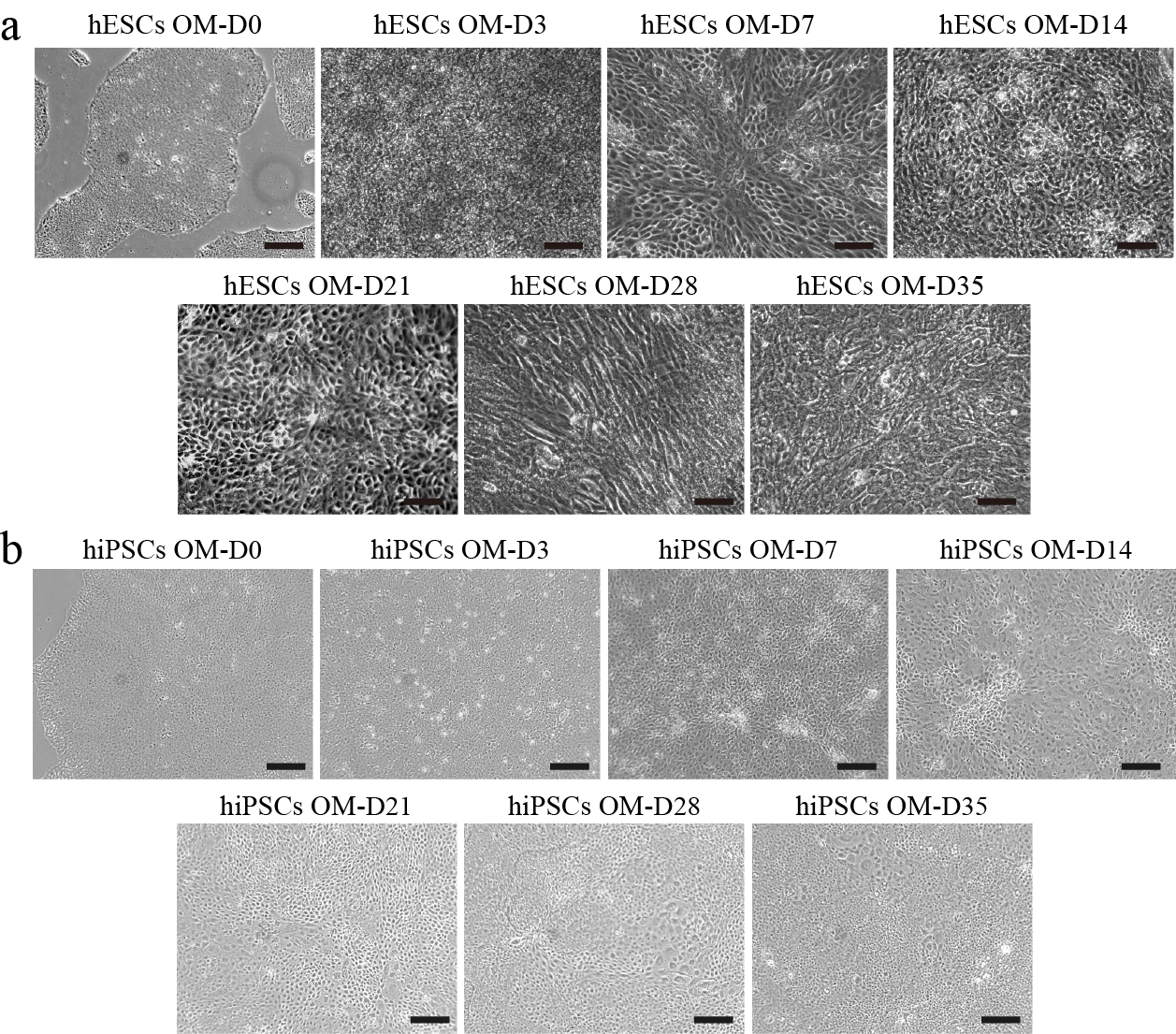


Figure S1. Cell morphology of H9 hESCs (a) and hNF-C1 hiPSCs (b) that cultured on Matrigel surface after culturing in serum-containing osteogenic induction medium for various periods (0 days, 3 days, 7 days, 14 days, 21 days, 28 days and 35 days). Scale bars, 200 μm.


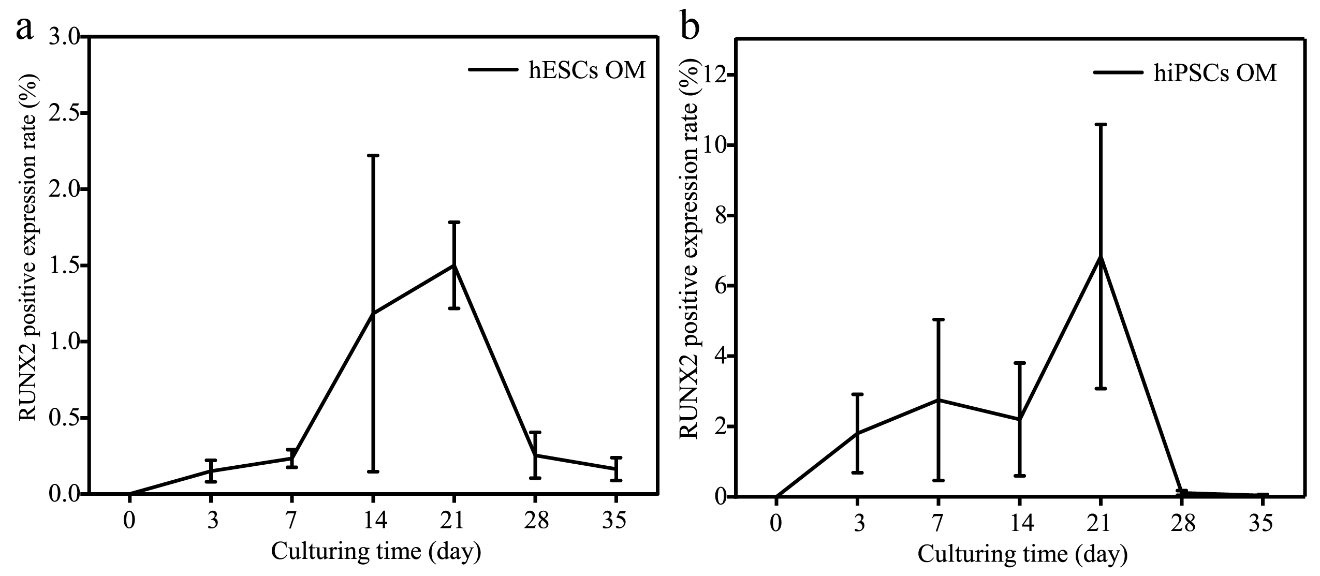


Figure S2. After osteogenic differentiation for different times (0 days, 3 days, 7 days, 14 days, 21 days, 28 days and 35 days), the number of DAPI stained cells and RUNX2-positive expressed cells were measured using an image J software for H9 hESCs (a) and hNF-C1 hiPSCs (b). For each well with stained samples, five images were obtained at 5 fixed positions (upper, lower, medium, left and right). The positive expression rate of RUNX2 protein was shown. n=3.
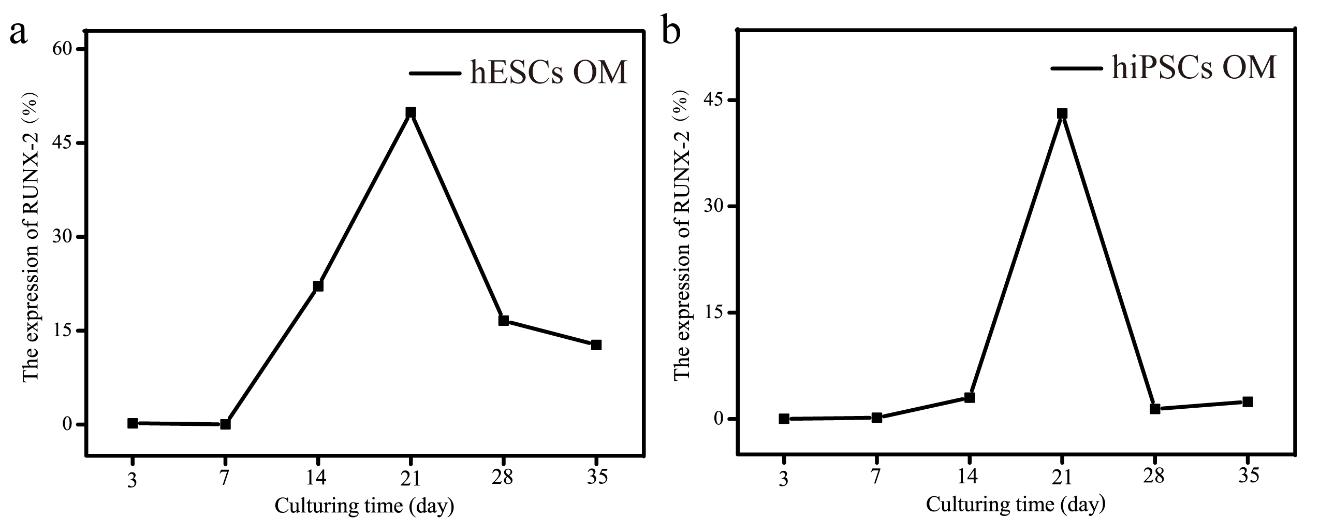


Figure S3. The positive expression rate of RUNX2 protein in H9 hESCs (a) and hNF-C1 hiPSCs (b) was detected by flow cytometry during osteogenic differentiation for different days (3, 7, 14, 21, 28 and 35).


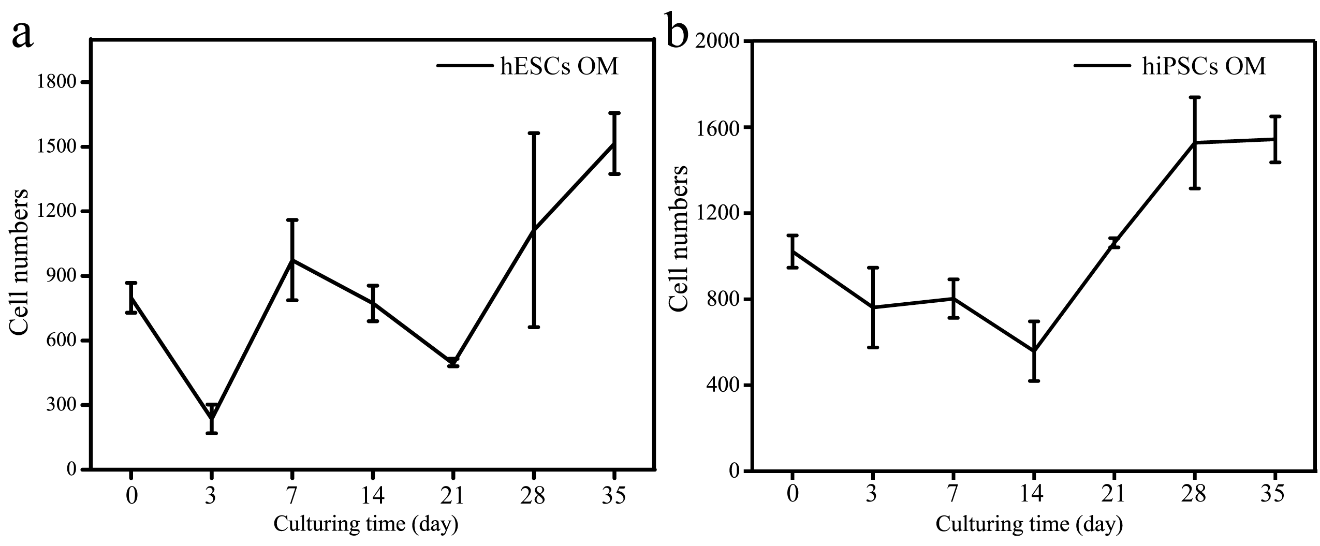


Figure S4. After osteogenic differentiation for different times (0 days, 3 days, 7 days, 14 days, 21 days, 28 days and 35 days), cell numbers of H9 hESCs (a) and hNF-C1 hiPSCs (b) were counted by DAPI staining. For each well with stained samples, five images were obtained at 5 fixed positions (upper, lower, medium, left and right). The number of cells stained cell nucleus were measured using imageJ software. n=3.


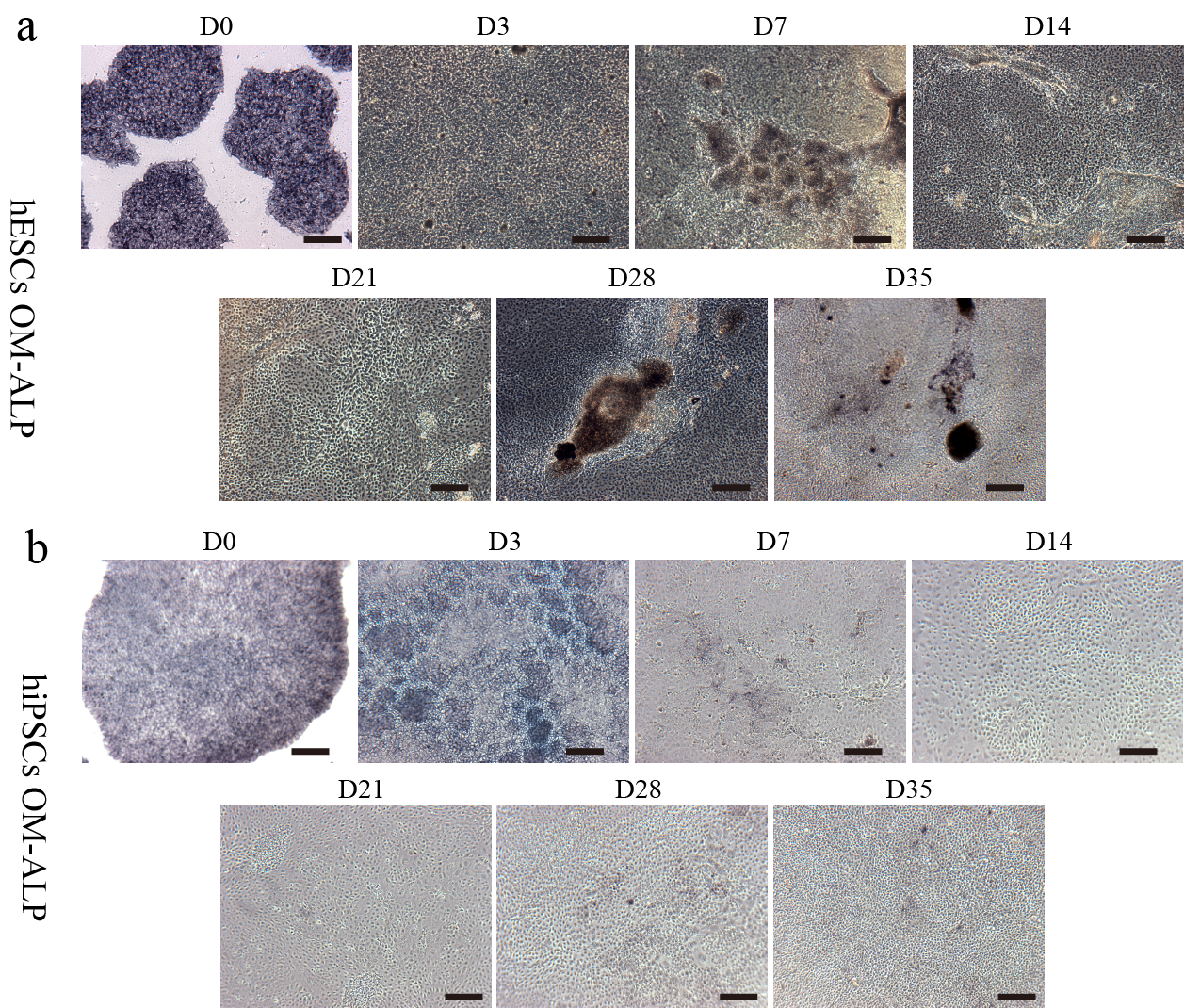


Figure S5. The ALP staining profile of H9 hESCs (a) and hNF-C1 hiPSCs (b) of the undifferentiated cell colonies as well as after during osteogenic induction for 3 days, 7 days, 14 days, 21 days, 28 days or 35 days. Scale bars, 200 μm.


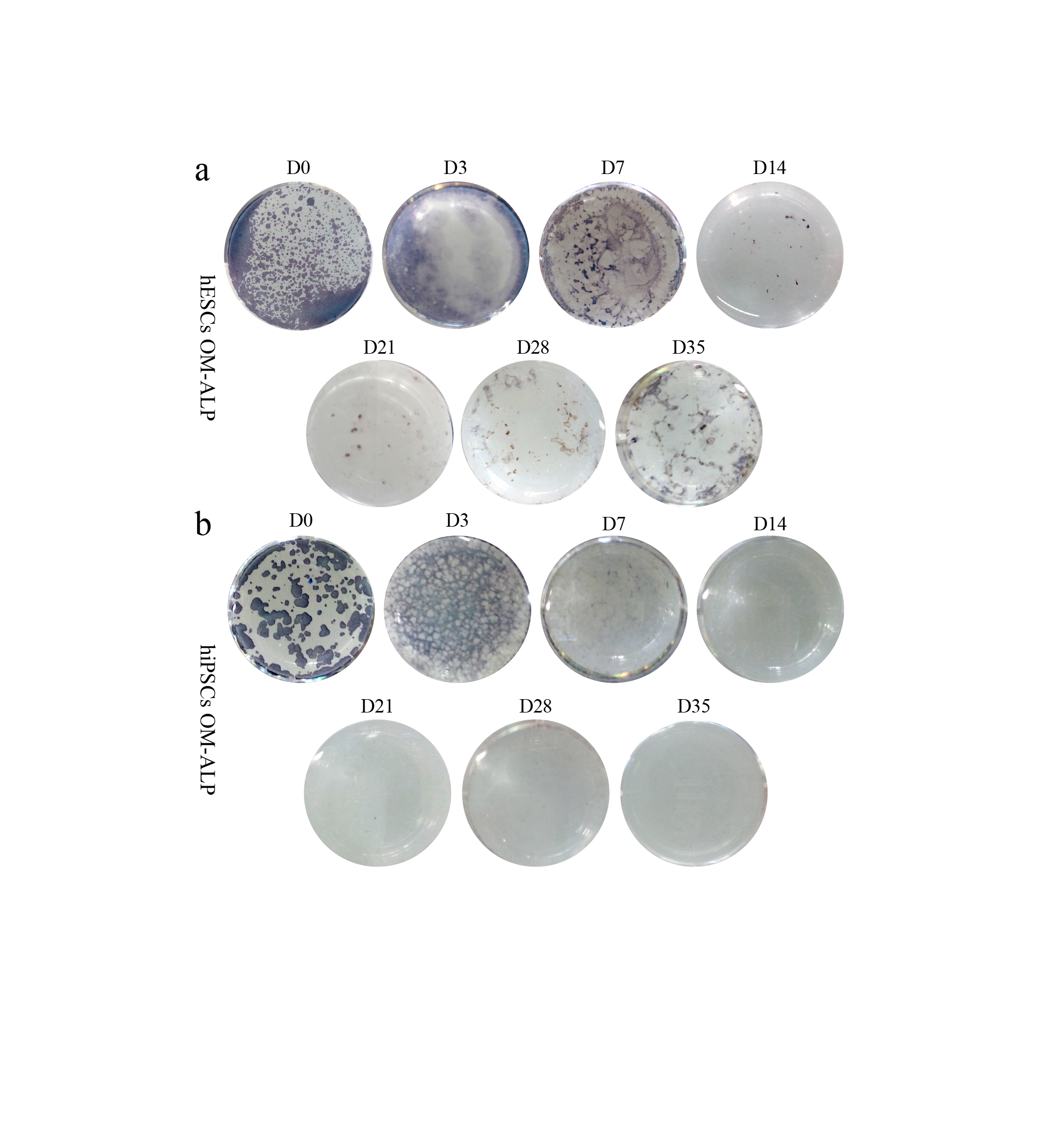


Figure S6. The ALP staining photograph of cell culture plates with H9 hESCs (a) and hNF-C1 hiPSCs (b) after culturing in osteogenic induction medium for different times (0 days, 3 days, 7 days, 14 days, 21 days, 28 days and 35 days).

Table S1. The sequences of the primers for RT-PCR.

| Primer |  | | Forward sequence | | | | Reverse sequence | |
| --- | --- | --- | --- | --- | --- | --- | --- | --- |
| *NANOG* |  | | TGAACCTCAGCTACAAACAG | | | | TGGTGGTAGGAAGAGTAAAG | |
| *ACTB* | | |  | | CCCAGAGCAAGAGAGG | | | GTCCAGACGCAGGATG |
| *OCT-4* | | |  | | CCTCACTTCACTGCACTGTA | | | CAGGTTTTCTTTCCCTAGCT |
| *TERT* | | |  | | GCCTTCAAGAGCCACGTC | | | CCACGAACTGTCGCATGT |
| *ALP* | | |  | | CAACCCTGGGGAGGAGAC | | | GCATTGGTGTTGTACGTCTTG |
| *COL1A1* | | |  | | GGGATTCCCTGGACCTAAAG | | | GGAACACCTCGCTCTCCA |
| *OCN* | |  | | TGAGACCCTCACACTCCTC | | ACCTTTGCTGGACTCTGCAC | | |
| *RUNX2* | |  | | GTGCCTAGGCGCATTTCA | | GCTCTTCTTACTGAGAGTGGAAGG | | |

Table S2. Antibodies applied for analysis of immunofluorescence.

| Testing |  | Antibodies | Source | Cat# | Dilution |
| --- | --- | --- | --- | --- | --- |
| OCT-3/4 | Primary antibodies | Mouse IgG anti-OCT-4 | Stem cell | 01550 | 1:100 |
| RUNX2 |  | Mouse IgG anti-RUNX2 | Abcam | ab76956 | 1:200 |
| OCN |  | Mouse IgG anti-OCN | Abcam | ab13418 | 1:200 |
| COL1A1 |  | Rabbit polyclonal anti-COL1A1 | Abcam | Ab34710 | 1:200 |
| OCT-3/4 | Second antibodies | Alexa Fluor 488 goat anti -mouse IgG | Invitrogen | A28175 | 1:500 |
| RUNX2/OCN/  COL1A1 |  | Alexa Fluor 488 goat anti -rabbit IgG | Life | A11008 | 1:500 |
